# Population pharmacokinetics of efpeglenatide in individuals with obesity and with type 2 diabetes

**DOI:** 10.3389/fphar.2025.1715585

**Published:** 2025-12-01

**Authors:** Seungchan Choi, Jiyoung Seo, Suemin Park, Na Young Kim, Hyeeun Kim, Hyeong-Seok Lim

**Affiliations:** 1 Asan Medical Center, Department of Clinical Pharmacology and Therapeutics, University of Ulsan College of Medicine, Seoul, Republic of Korea; 2 Asan Medical Center, Department of Medical Science and Asan Medical Institute of Convergence Science and Technology, University of Ulsan College of Medicine, Seoul, Republic of Korea; 3 Hanmi Pharm. Co., Ltd., Seoul, Republic of Korea

**Keywords:** efpeglenatide, HM11260C, GLP-1 receptor agonist, populationpharmacokinetics, obesity, modeling and simulation, dose optimization, NONMEM

## Abstract

**Background:**

Efpeglenatide (HM11260C) is a long-acting GLP-1 receptor agonist under development for obesity. A population pharmacokinetic (PK) analysis was conducted to characterize its PK properties and evaluate covariate effects to support clinical dosing strategies.

**Methods:**

Pooled PK data from six clinical studies in participants with type 2 diabetes or obesity were analyzed using nonlinear mixed-effects modeling (NONMEM). Covariate effects, model diagnostics, and simulations were used to assess exposure and dosing strategies.

**Results:**

A two-compartment model with dual absorption pathways adequately described the data. Body weight and disease status influenced absorption and clearance; however, predicted exposure differences across weight percentiles and demographic subgroups were modest and within conventional bioequivalence limits. Simulations suggested approximately dose-proportional increases across the evaluated dose range with once-weekly administration and supported the feasibility of stepwise dose-escalation.

**Conclusion:**

Efpeglenatide PK was well characterized across type 2 diabetes and obesity populations. Although some covariates affected PK parameters, their impact on exposure was not clinically meaningful. These results support a uniform dosing strategy without routine dose adjustment and provide quantitative evidence for stepwise dose escalation in ongoing clinical development for obesity.

## 1 Introduction

Glucagon-like peptide-1 (GLP-1) is an incretin hormone secreted by intestinal L cells in response to nutrient intake (Holst, 2007). It enhances glucose-dependent insulin secretion, suppress glucagon release, delay gastric emptying, and promote satiety (Müller et al., 2019). These actions are associated with improved glycemic control and reduced in body weight (Drucker, 2022). However, native GLP-1 is rapidly degraded by dipeptidyl peptidase-4 (DPP-4), which limits its therapeutic applicability (Holst, 2007). To overcome this limitation, GLP-1 receptor agonists (GLP-1 RAs) have been developed to provide prolonged receptor activation and are now widely used for managing metabolic diseases, including obesity (Drucker, 2022; Zheng et al., 2024).

Efpeglenatide (HM11260C) is a long-acting GLP-1 RA currently under clinical development for the treatment of obesity (Reid et al., 2019). It is a CA-exendin-4 analogue covalently linked to the Fc fragment of human immunoglobulin G4 via a non-peptidyl linker (LAPSCOVERY® platform). This structural modification reduces clearance and prolongs its half-life, thereby allowing once-weekly or potentially once-monthly administration (Yoon et al., 2020; Rosenstock et al., 2019). Clinical studies have shown that efpeglenatide leads to reductions in body weight and improvements in glycemic parameters, with these effects maintained even at extended dosing intervals (Pratley et al., 2019; Del Prato et al., 2020; Rosenstock et al., 2019). Nevertheless, clinical trial data alone provide limited insight into the variability in drug exposure and the quantitative influence of intrinsic factors such as body weight. Given its long half-life and the potential for flexible dosing regimens, a population pharmacokinetic (PK) analysis is warranted to characterize the PK of efpeglenatide, assess covariate effects, and explore dosing strategies for the treatment of obesity.

In this study, a population PK analysis of efpeglenatide was performed using pooled PK data from one phase 1 study and five phase 2 studies that, included patients with type 2 diabetes mellitus (T2DM) and non-diabetic individuals with obesity (Yoon et al., 2020; Rosenstock et al., 2019; Pratley et al., 2019; Del Prato et al., 2020; Hompesch et al., 2021; Escobar et al., 2023). The primary objective was to characterize the PK of efpeglenatide and to evaluate the influence of clinically relevant covariates, such as body weight, on drug exposure. Model-based simulations were also performed predict plasma concentrations over time following various dosing regimens including stepwise dose-escalation, and to evaluate exposure across subgroups, thereby providing quantitative evidence to support the ongoing clinical development of efpeglenatide for the treatment of obesity.

## 2 Materials and methods

### 2.1 Study population

The population PK analysis was performed based on pooled data from one phase 1 and five phase 2 studies of efpeglenatide. The phase 1 study (HM-EXC-102) was an exploratory, randomized, partially single-blind trial. The Phase 2 studies (HM-EXC-201, -202, −203, −204, and −205) were randomized, double-blind, placebo-controlled or open-label designs. Liraglutide comparator groups in HM-EXC-102 and HM-EXC-203 were excluded from the analysis. Dosing regimens ranged from once weekly (QW) to every other week (Q2W) and once monthly (QM) subcutaneous administration. Key study characteristics are presented in Table 1. The key demographic and clinical characteristics of the study populations are summarized in Table 2.

**TABLE 1:** Key design elements for clinical studies included in the population PK analysis.

Study	Phase	Design	Treatment^a^	Population
HM-EXC-102	1	An exploratory, randomized, partially single-blind, placebo- and open-label- controlled, parallel group study designed to assess the effects of efpeglenatide or liraglutide on gastric emptying and beta-cell response in subjects with T2DM	• Group A 6 mg efpeglenatide or placebo QW^b^ • Group B 16 mg efpeglenatide or placebo QM^b^	T2DM Approximately 16 subjects in group A and group B (12 active and 4 placebo)
HM-EXC-201	2	A double blind, randomized, placebo controlled, multiple ascending dose study to determine the tolerability, pharmacokinetics and pharmacodynamics of efpeglenatide in T2DM subjects on stable metformin monotherapy	Single dose • Efpeglenatide^b^ (2, 4, 8, 14, 20, 40, 60 and 100 μg/kg)	T2DM 6 subjects per group (5 active, 1 placebo)
HM-EXC-202	2	A double-blind, randomized, placebo- controlled, multiple ascending-dose trial to determine the tolerability, pharmacokinetics, and pharmacodynamics of the GLP-1 receptor agonist efpeglenatide in adult subjects with T2DM on stable metformin therapy	• Weekly groups^b^ W1: 1 mg or placebo QW W2 : 2 mg or placebo QW W3 : 4 mg or placebo QW • Monthly groups^b^ M1: 8 mg or placebo QM M2: 12 mg or placebo QM M3: 16 mg or placebo QM	T2DM Approximately 12 subjects in each group (9 active, 3 placebo)
HM-EXC-203	2	A 12-week, double-blind, randomized, parallel-group, multicenter, international trial to assess the effect of glycemic control of five doses of efpeglenatide versus placebo or open-label liraglutide in subjects with type 2 diabetes	• Placebo • Efpeglenatide^b^ (0.3, 1, 2, 3, or 4 mg QW)	T2DM Approximately 30 subjects per treatment arm
HM-EXC-204	2	A 16-week, double-blind, placebo-controlled, parallel group, randomized, multicenter study designed to assess the effect of three different monthly subcutaneous doses of efpeglenatide or placebo on glycemic control in subjects with inadequately controlled T2DM receiving stable dosages of metformin	• Placebo • Efpeglenatide^b^ (8 mg, 12 mg, or 16 mg QM)	T2DM Approximately 50 subjects per treatment arm
HM-EXC-205	2	A double-blind, randomized, placebo- controlled, multiple ascending-dose trial to determine the tolerability, pharmacokinetics, and pharmacodynamics of the GLP-1 receptor agonist efpeglenatide in adult subjects with T2DM on stable metformin therapy	Efpeglenatide^b^ (4 mg QW, 6 mg QW 6 mg Q2W, and 8 mg Q2W)	Obesity Approximately 54 subjects per treatment arm

^a^ In HM-EXC-102, and HM-EXC-203, liraglutide was also included in the control group; however the dosing regimen is not detailed in this report as these groups were excluded from the modeling and simulation analyses.

^b^ Administered subcutaneously in the abdomen.

T2DM, type 2 diabetes mellitus; QW, once a week; QM, once a month; QD, once daily; GLP-1, glucagon-like peptide-1; Q2W, every other week.

Data are summarized from previous efpeglenatide trials (Pratley et al., 2019; Rosenstock et al., 2019; Yoon et al., 2020; Del Prato et al., 2020; Hompesch et al., 2021).

**TABLE 2:** Summary of subject characteristics of efpeglenatide clinical studies included in population PK analysis.

Study number	HM-EXC-102	HM-EXC-201	HM-EXC-202	HM-EXC-203	HM-EXC-204	HM-EXC-205	Total
Number of subjects (%)	26 (5.2)	40 (8.0)	51 (10.2)	81 (16.3)	95 (19.1)	205 (41.2)	498 (100)
Characteristics							
Continuous variable	Median (min–max)						
Age (years)	54 (27–66)	63 (47–75)	54 (26–65)	58 (26–74)	58 (30–74)	44 (18–64)	52 (18–64)
Weight (kg)	91.1 (52.5–128.7)	92.3 (68.2–121.9)	97.3 (59.8–153)	91.8 (49.7–133.5)	88 (58.6–152)	96.6 (64.4–191)	93.6 (49.7–191)
BMI (kg/m^2^)	32.5 (23.4–44.7)	28.8 (24.8–39.7)	32.7 (26.2–44.5)	32.7 (19.2–40.3)	31.6 (24.2–43.3)	35.1 (28.1–57.7)	33.4 (19.2–57.7)
LBW	59.1 (37.4–80)	66.8 (44–77.7)	62.6 (40.8–82.9)	59.2 (38.8–82.7)	56.9 (40.4–89.4)	52.6 (34.7–87.6)	55.1 (34.7–89.4)
Height (cm)	166.2 (147.5–185.5)	176 (153–193)	170.2 (149.9–190.5)	168 (145–194)	168 (148–201)	166 (142–193)	168 (142–201)
Categorical variable	Number of subjects (%)						
Gender							
Male	15 (6.7)	34 (15.2)	28 (12.6)	46 (20.46)	52 (23.3)	48 (21.5)	223 (44.78)
Female	11 (4.0)	6 (2.2)	23 (8.4)	35 (12.7)	43 (15.6)	157 (57.1)	275 (55.22)
Population							
T2DM patients	26 (8.9)	40 (13.7)	51 (17.4)	81 (27.6)	95 (32.4)	0 (0)	293 (58.84)
Obesity	0 (0)	0 (0)	0 (0)	0 (0)	0 (0)	205 (100)	205 (41.16)
Race							
Caucasian	21 (5.3)	35 (8.8)	40 (10.1)	77 (19.3)	85 (21.4)	140 (35.2)	398 (79.92)
Black	3 (4.6)	0 (0)	11 (16.9)	4 (6.2)	8 (12.3)	39 (60.0)	65 (13.05)
Asian	0 (0)	2 (8.7)	0 (0)	0 (0)	1 (4.3)	20 (87.0)	23 (4.62)
Native Hawaiian or pacific islander	0 (0)	0 (0)	0 (0)	0 (0)	0 (0)	2 (100.0)	2 (0.4)
Others	2 (20.0)	3 (30.0)	0 (0)	0 (0)	1 (10.0)	4 (40.0)	13 (1.92)

BMI, body mass index; LBW, lean body weight; T2DM, type 2 diabetes mellitus.

All clinical studies were conducted in accordance with the Declaration of Helsinki, the International Council for Harmonisation (ICH) E6 (R2) Good Clinical Practice guidelines, and all applicable local regulatory requirements. Study protocols were reviewed and approved by the Institutional Review Board or Independent Ethics Committee at each participating center, and written informed consent was obtained from all participants prior to enrollment.

### 2.2 Dataset and measurements

The pooled dataset comprised six clinical studies (five studies in patients with T2DM and one study in obesity). A total of 3,596 plasma efpeglenatide concentration measurement were collected from 498 subjects across six clinical studies at prespecified time points.

All samples were analyzed at certified laboratories using validated analytical method. Serum concentrations of efpeglenatide were quantified using a validated Enzyme-Linked Immunosorbent Assay (ELISA) method.

For the PK analysis, 3,316 post-dose plasma concentrations above the lower limit of quantification (LLOQ) were used in the model estimation. All pre-dose concentrations before the first administration were excluded from the dataset. Post-dose samples below the limit of quantification (BLQ) were treated as missing (i.e., not included in the analysis). In total, 117 pre-dose samples (3.25%) and 153 post-dose samples (4.0%) were BLQ.

### 2.3 Population PK modeling strategy and structural model

**FIGURE 1:**
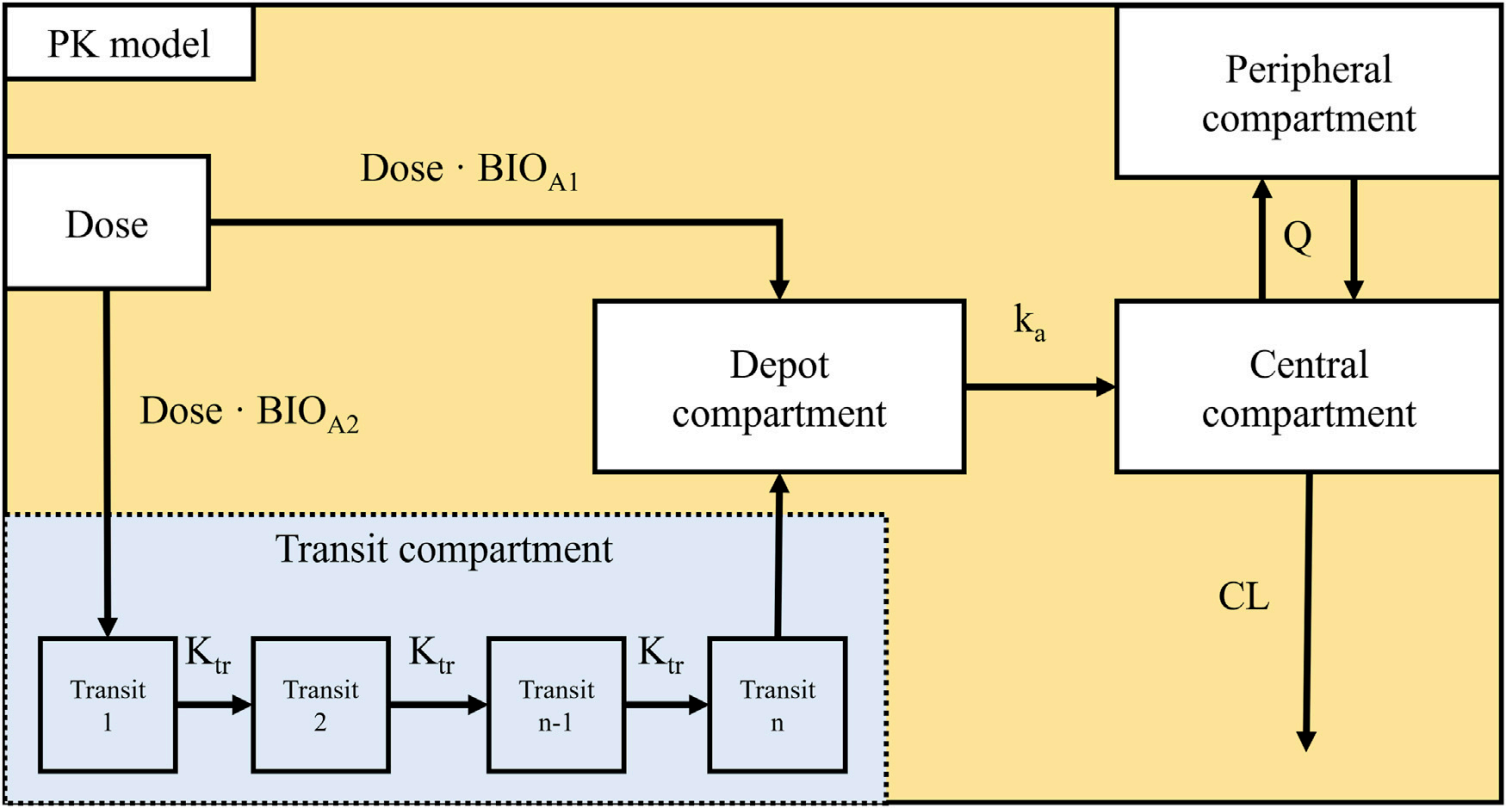
Diagram of the Final PK model Abbreviation: ka is the absorption rate constant, K_tr_ is the transit rate constant from the nth-1 compartment to the *n*th compartment and is calculated as $\frac{n+1}{MTT}$ , MTT is the mean transit time, n is the number of transit compartments, V_C_ is the apparent central volume of distributions, V_P_ is the peripheral volume of distribution, Q is the inter-compartment clearance, and CL is the clearance.

The base PK model evaluation was begun with a one-compartment disposition model with first-order elimination, with additional refinement to better characterize the absorption phase. To describe the double peaks observed during the absorption phase, an empirical absorption model incorporating two divided subcutaneous administrations was implemented. The first one of the two divided doses was described as the first order absorption after bolus doses into depot compartment, capturing the first peak during the absorption phase. The second part of the dose was introduced to describe the second peak with time delay during absorption phase, which was described using a transit compartment model (Savic et al., 2007). This dual dose approach successfully described the double-peak observed in the PK data, and significantly improving model fit. After the absorption model was established, various compartment models were evaluated. Ultimately, a two-compartment disposition model with first-order elimination was selected as the base PK model (Figure 1).

The transit compartment model describes the delay in absorption as the sequential passage of the drug through a chain of transit compartments, each connected by a first-order transit rate constant (K_tr_). The number of transit compartments (n) and the mean transit time (MTT) were estimated from the data, with K_tr_ calculated as (n+1)/MTT. Drug exiting the final transit compartment entered the absorption compartment and was transferred to the central compartment via first-order absorption, as described by Equations 1–3. (Savic et al., 2007).

$$\frac{da_{n}}{dt}=K_{tr}\cdot a_{\left(n-1\right)}-K_{tr}\cdot a_{n}$$ (1)

$$a_{n}\left(t\right)=\text{Dose}\cdot F\cdot \frac{{\left(K_{tr}\cdot t\right)}^{n}}{n!}\cdot e^{-K_{tr}\cdot t}$$ (2)

$$n!\approx \sqrt{2\pi }\cdot n^{n+0.5}\cdot e^{-n}$$ (3)

The amount of drug administered subcutaneously (A_sc_) in the depot, central compartment (A_c_) and peripheral compartment (A_p_) were described using the following differential Equations 4–6:

$$\frac{dA_{\text{sc}}}{\text{dt}}=\text{Dose}\cdot {BIO}_{A1}+\text{Dose}\cdot {BIO}_{A2}\cdot \frac{{\left(K_{\text{tr}}\cdot t\right)}^{n}\cdot e^{-K_{\text{tr}}\cdot t}}{\sqrt{2\pi }\cdot n^{n+0.5}\cdot e^{-n}}-k_{a}\cdot A_{\text{sc}}$$ (4)

$$\frac{dA_{c}}{\text{dt}}=k_{a}\cdot A_{\text{sc}}-\frac{A_{c}}{V_{c}}\cdot \text{CL}-\frac{A_{c}}{V_{c}}\cdot Q+\frac{A_{p}}{V_{p}}\cdot Q$$ (5)

$$\frac{dA_{p}}{\text{dt}}=\frac{A_{c}}{V_{c}}\cdot Q-\frac{A_{p}}{V_{p}}\cdot Q$$ (6)

To confine the estimated fraction values to the range between 0 and 1 and to allow implementation of interindividual variability, a logit transformation was applied. BIO_A1_ and BIO_A2_ represent the fractions of the administered dose entering the depot compartment via the bolus administration route and the delayed absorption route, respectively. The transformation was defined as shown in Equation 7 (Adedokun et al., 2020):

$${BIO}_{A1}=\frac{e^{BIOF}}{\left(1+e^{BIOF}\right)},{BIO}_{A2}=\frac{1}{\left(1+e^{BIOF}\right)}$$ (7)

where BIOF is an unbounded parameter ( −∞∼∞ ) representing the logit-transformed value of BIOA1, and BIO_A2_ = 1-BIO_A1_

The population PK modeling analysis was performed using NONMEM version 7.5 (ICON Development Solutions, Hanover, MD, United States). The first-order conditional estimation method with interaction (FOCEI) was used to approximate the marginal likelihood and facilitate estimation of population model parameters. Pre- and post-processing of the data and analysis of modeling results were performed using R software version 4.3.1 or other (R Core Team, 2023).

### 2.4 Statistical model

Interindividual variability (IIV) in most PK parameters was modeled using an exponential distribution (Equation 8). For the *i*th individual, *η*_*i*_, is a random variable assumed to be independently selected from normal distribution with a mean of zero and variance of ω^2^.

$$P_{i}=P_{TV}\times \exp\left(\eta_{i}\right)$$ (8),

where $P_{TV}$ is the typical value (population value) of the fixed effect parameter. The approximate percent coefficient of variation (%CV) was calculated as shown in Equation 9:

$$\%CV\left(IIV\right)=\sqrt{\exp\left(\omega^{2}\right)-1}\times 100$$ (9)

Residual variability (RV), representing within-subject unexplained variability and contributions from assay error, sampling deviations, and model misspecification, was evaluated using additive, proportional, and combined residual error models. Model selection was guided by the likelihood ratio test (LRT) and inspection of goodness-of-fit (GOF) plots, and the proportional error model was selected as the final residual error structure (Equation 10). The RV for the *j*th observed value in the *i*th individual and ε_prop,ij_; represents the proportional component, assumed to be independent and normally distributed, with a mean of zero and a variance of σ^2^.

$$Y_{ij}={IPRED}_{ij}\times \left(1+\varepsilon_{prop,ij}\right)$$ (10)

where y_ij_: *j*th observed value in the *i*th individual; IPRED_ij_: *j*th model predicted value for the *i*th individual; ε_prop,ij_ is the proportional error.

### 2.5 Model selection and evaluation

Model selection and evaluation were performed using both statistical and graphical approaches. For statistical evaluation, the LRT was used to compare hierarchical models, with a p-value <0.05 (corresponding to a decrease in objective function value [OFV] of 3.84 points) considered statistically significant, assuming that the difference in −2 log-likelihood (-2LL) follows an approximate chi-square distribution (Karlsson et al., 1998). In addition, the Wald test was used to assess parameter significance and precision: 95% confidence intervals (CIs) for each parameter estimate were constructed using the point estimate and standard error derived from the model outcomes, and parameters were considered statistically significant if the 95% CI excluded zero (Hooker et al., 2007).

Graphical evaluation included the inspection of standard GOF plots and visual predictive checks (VPCs) to compare observed versus model-predicted values, with predictive performance assessed in terms of both central tendency and variability (Savic et al., 2007). VPCs were performed by generating 1000 replicates using Monte Carlo simulations in NONMEM, and the resulting simulated prediction intervals were overlaid on the original PK data in plots generated using R. In addition, prediction-corrected VPCs (pcVPCs) stratified by key covariates (body weight and disease status) were performed to assess model performance across subgroups (Karlsson and Savic, 2007; Beal et al., 2009).

The stability of parameter estimates for all fitted models was assessed by examining pairwise correlations (with ρ > 0.95 indicating potential collinearity) in the correlation matrix and evaluating the condition number (ratio of the largest to smallest eigenvalues), with values below 1000 considered acceptable (Jonsson and Karlsson, 1998).

### 2.6 Covariate assessment

Covariate selection was performed using the stepwise covariate modeling (SCM) approach guided by the LRT. In the forward inclusion step, each covariate–parameter relationship was tested individually, and the covariate was retained if the decrease in the OFV (ΔOFV) was ≥7.87 (p < 0.005, χ^2^ distribution with 1 degree of freedom). In the subsequent backward elimination step, starting from the full model, covariates were sequentially removed if the increase in OFV upon removal was <10.8 (p < 0.001). The covariates evaluated included body weight, age, sex, race (Caucasian, Black, Asian, Native Hawaiian or Pacific Islander, and Others), body mass index (BMI), lean body weight (LBW), and obesity status. All covariates were tested for potential influence on all PK parameters (Jonsson and Karlsson, 1998).

Continuous covariates were incorporated using a power function normalized to the reference (median) value, as shown in Equation 11:

$${TVP}_{i}={TVP}_{REF}{\left(\frac{{COV}_{i}}{{COV}_{REF}}\right)}^{\theta }$$ (11)

where ${\text{TVP}}_{\text{REF}}$ and θ are fixed-effect parameters and ${\text{COV}}_{\text{REF}}$ is a reference value of covariate ${\text{COV}}_{i}$ . For this analysis, the approximate median of the population will be used for ${\text{COV}}_{\text{REF}}$ .

Categorical covariates were modeled using an exponential/indicator function, as shown in Equation 12:

$${\text{TVP}}_{i}={\text{TVP}}_{\text{REF}}\cdot \left(1-{\text{Cat}}_{i}\right)+{\text{TVP}}_{Cat}\cdot {\text{Cat}}_{i}$$ (12)

where ${\text{TVP}}_{\text{REF}}$ and ${\text{TVP}}_{Cat}$ are fixed-effect parameters without and with the covariate, respectively. ${\text{Cat}}_{i}$ is an indicator variable that takes a value of 0 or 1 depending on whether the subject has the covariate or not.

After final model selection, the numerical stability of parameter estimates (e.g., standard errors [SE] and relative standard errors [RSE]) was evaluated, and covariates associated with unstable estimates were excluded.

Inter-occasional variability (IOV) in PK was assessed by introducing a random effect parameter to selected fixed effect PK parameters, allowing different random value to assigned for each dosing occasion within the same individual.

### 2.7 Model based simulation

Monte Carlo simulations of plasma efpeglenatide concentration–time profiles were performed using NONMEM using the final population PK model. Each scenario consisted of 1,000 virtual subjects receiving the specified dosing regimen, and was designed to evaluate steady-state exposure and the impact of stepwise dose escalation on PK profiles. IIV and RV were incorporated to reflect population variability. Covariates were fixed based on predefined subgroup scenarios rather than sampled from distributions.

## 3 Result

### 3.1 PK model

The PK of efpeglenatide was well-described by a two-compartment linear model with dual absorption pathways, which characterized the pooled data from six completed clinical studies (Table 3). IIV was estimated for all parameters except Q/F and N, with no statistically significant covariance identified between IIV terms. IOV was evaluated but not found to be significant and was therefore not included in the final model.

**TABLE 3:** Final population pharmacokinetic parameter estimates of efpeglenatide.

Parameter (units)	Definition	Estimate [%RSE]	Inter-individual variability [%RSE]
k_a_ (1/h)	Absorption rate constant	0.006 [4.33]	21.53 [28.48]
Covariate effect (θ) of body weight on k_a_	Covariate effect (θ) in the power model is described as ${\left(k_{a}\right)\times \left(\frac{WT}{92}\right)}^{\theta }$	−0.927 [14.24]	-
V_C_/F (L)	Volume of the central compartment	2.80 [7.39]	68.31 [18.28]
V_P_/F (L)	Volume of the peripheral compartment	3.96 [6.57]	24.97 [27.11]
Q/F (L/h)	Inter-compartment clearance	0.073 [11.14]	-
CL/F in obesity (L/h)	Clearance for obesity	0.032 [1.79]	23.01 [5.60]
CL/F in T2DM (L/h)	Clearance for patients with T2DM	0.044 [1.50]	
Covariate effect (θ) of body weight on CL/F	Covariate effect (θ) in the power model is described as ${\left(V_{C}/F\right)\times \left(\frac{WT}{92}\right)}^{\theta }$	0.964 [6.75]	-
MTT (hour)	Mean transit time	2.680 [13.47]	46.27 [37.14]
N	Number of transit compartments	5.520 [22.28]	-
BIOF	Logit parameter expressing the fraction of dose absorbed via the first-order and transit pathway	−1.11 [27.59]	-
BIO_A1_	Dose fraction absorbed via the first-order pathway	0.248^a^	66.93 [14.6]
BIO_A2_	Dose fraction absorbed via the transit-compartment pathway	0.752^b^	-
Residual error			
ε_prop_	Proportional error	0.15 [0.48]	-

Inter-individual variability (IIV) is expressed as the coefficient of variation (CV %); T2DM, type 2 diabetes mellitus; RSE, relative standard error; WT, body weight.

^a^ ${\text{BIO}}_{A1}\;was\;calculated\;as\;\frac{\exp\left(BIOF\right)}{1+\exp\left(BIOF\right)}$

^b^ ${\text{BIO}}_{A2}was\;derived\;as\;1-{\text{BIO}}_{A1}$

All fixed-effect PK parameters and random effect parameters for IIV were estimated with acceptable precision, with relative standard errors (RSEs) < 30% for all fixed effects. Although the IIV of Vc/F (CV: 68.31%, RSE: 18.28%), BIOA1 (CV: 66.93%, RSE: 14.6%), and MTT (CV: 46.27%, RSE: 35.88%) was relatively large, the corresponding RSE values suggested that these estimates were supported by the data. There was no sign of over-parameterization.

Covariate analysis revealed that body weight influenced both k_a_ and CL/F through a power model, with estimated exponents of −0.927 and 0.964, respectively. The disease status (T2DM vs. obese) also appeared to influence CL/F, with estimated values of 0.044 and 0.032, respectively.

**FIGURE 2:**
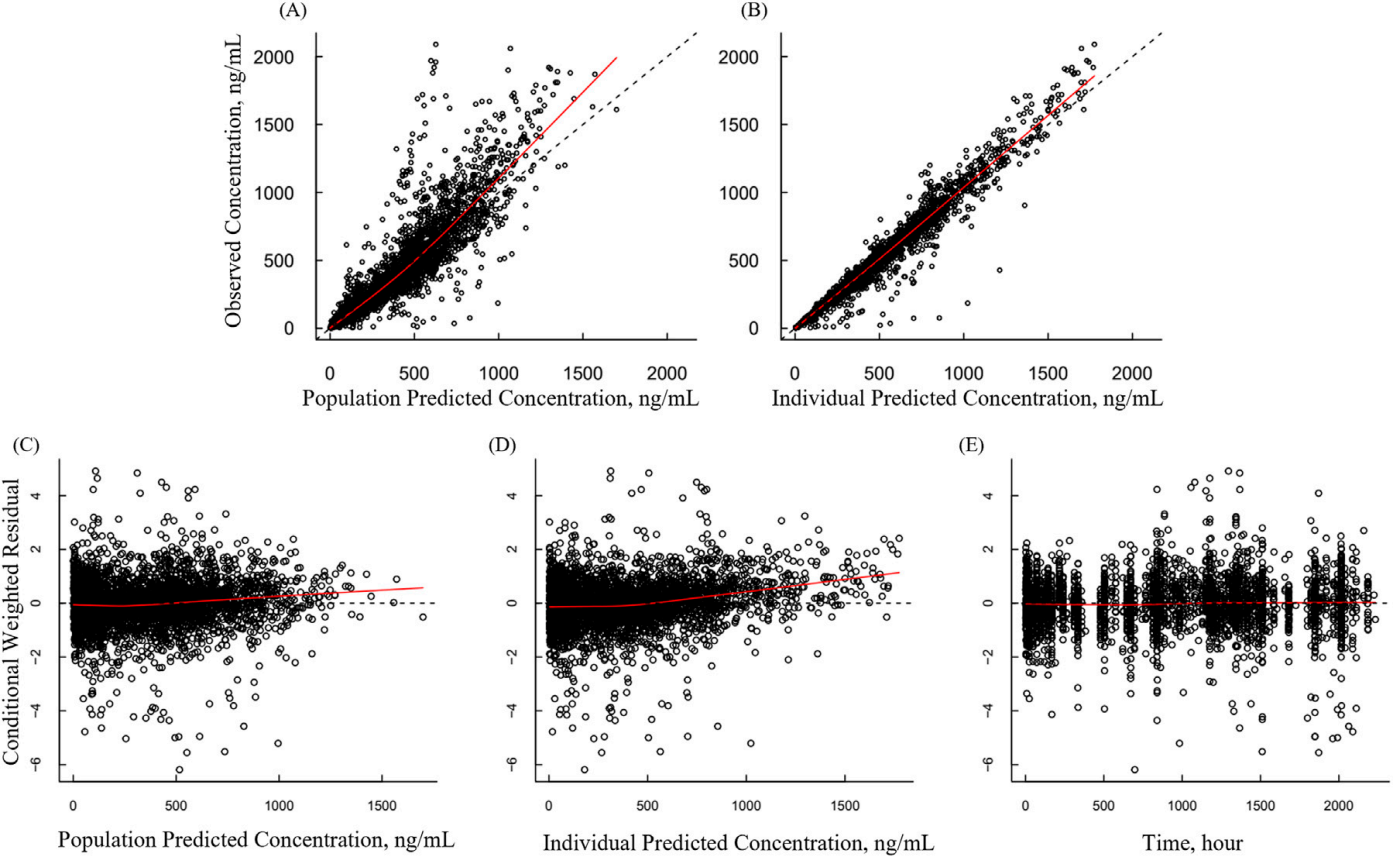
Basic goodness-of-fit plots for the final pharmacokinetic model **(A)** Observed versus population-predicted concentration; **(B)** observed versus individual-predicted concentration; **(C)** conditional weighted residuals (CWRES) versus population-predicted concentration; **(D)** CWRES versus individual-predicted concentration; **(E)** CWRES versus time. Black circles represent observed data, dashed lines represent the line of identity, and solid red lines represent the locally weighted scatterplot smoothing (LOWESS) fit.

Goodness-of-fit (GOF) plots for the PK model showed good agreement between observed and predicted plasma concentration (Figure 2A). Both population predicted and individual predictions versus observed concentrations showed that the data were randomly distributed around the line of identity, indicating no significant bias (Figure 2B). Residual diagnostics further supported model adequacy, as conditional weighted residuals were randomly distributed around zero across both the time and predicted concentration axes, no apparent trends or patterns observed (Figures 2C–E).

**FIGURE 3:**
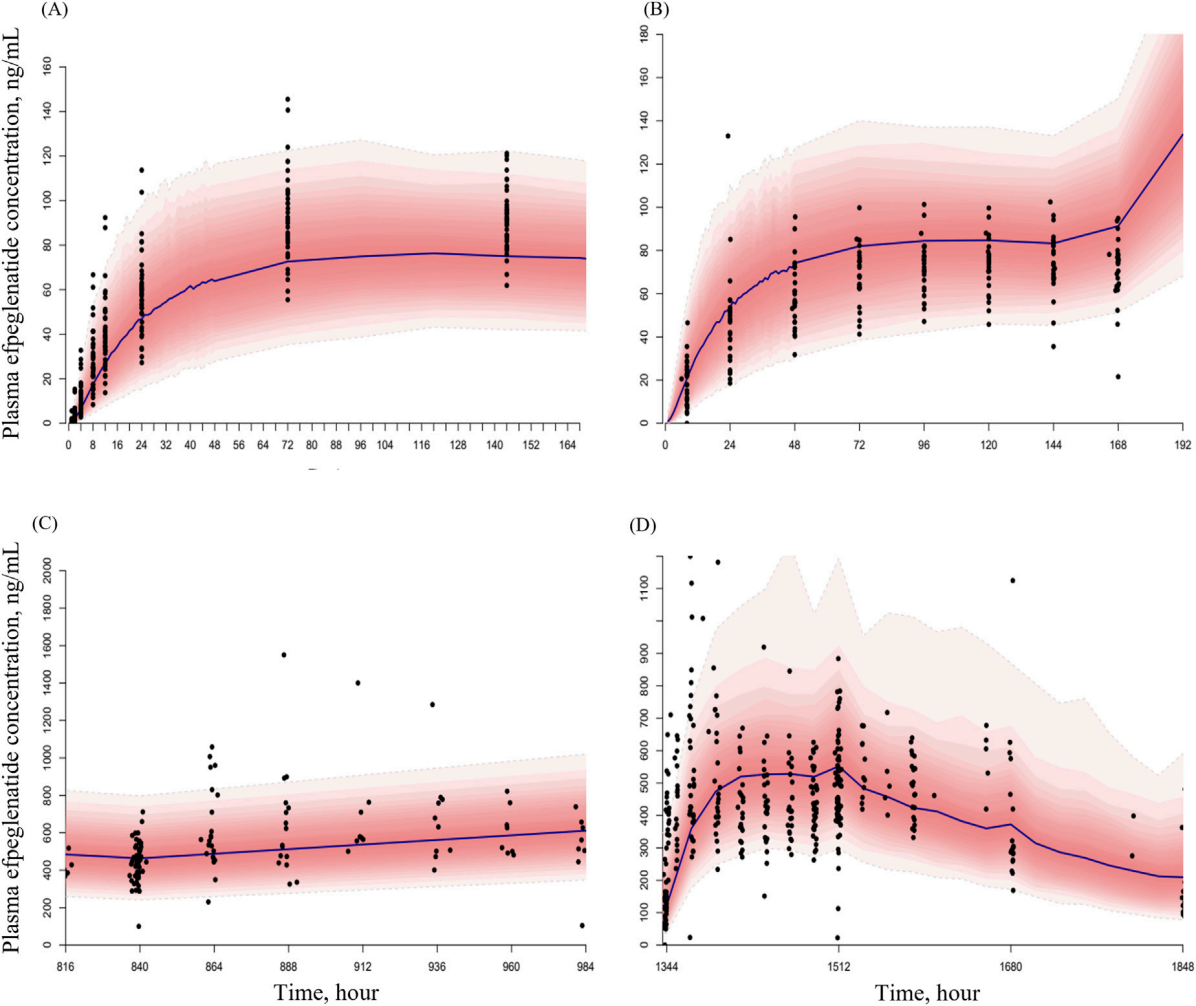
Prediction-corrected visual predictive checks (pcVPCs) for the final pharmacokinetic model of efpeglenatide across dosing regimens **(A)** single dose (patients with T2DM); **(B)** once weekly, QW (patients with T2DM and obesity); **(C)** once every 2 weeks, Q2W (patients with T2DM and obesity); and **(D)** once every 4 weeks, Q4W (patients with T2DM). Black circles represent observed concentrations, black solid lines represent the median model prediction, and red shaded area represents the 95% prediction intervals based on 1000 simulations.

The pcVPC for the final PK model showed good agreement between observed and predicted efpeglenatide concentration across dosing regimen. Approximately 95% of the observed values fell within the 95% prediction intervals, indicating that the model described an adequate description of the data (Figure 3).

### 3.2 Model-based simulation

**FIGURE 4:**
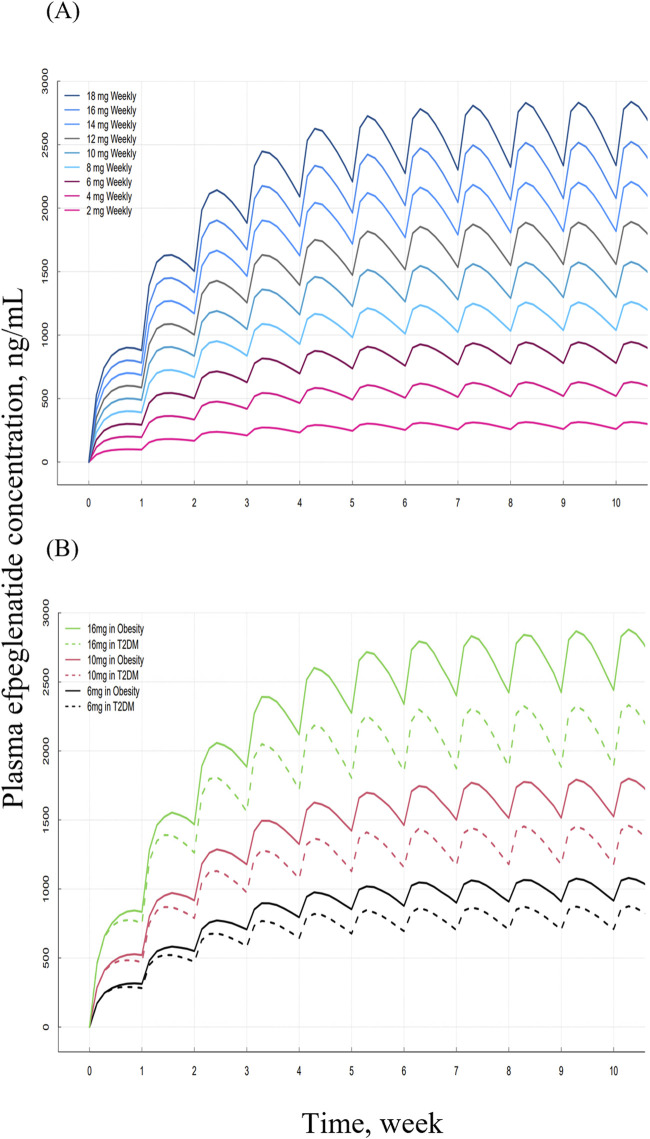
Simulation plasma efpeglenatide concentration-time profiles under different dosing regimens **(A)** Once-weekly dosing across the 2–18 mg range. **(B)** Comparison between subgroups of subjects with type 2 diabetes mellitus (T2DM) and obesity following once-weekly dosing 6, 10, 16 mg.

Model-predicted steady-state AUCs of efpeglenatide appeared to increase in a dose-proportional manner across the 2–18 mg once-weekly dosing range (Figure 4A). In subgroups analyses, obese subjects exhibited approximately 21% higher steady-state AUC compared to subjects with T2DM (Figure 4B).

**FIGURE 5:**
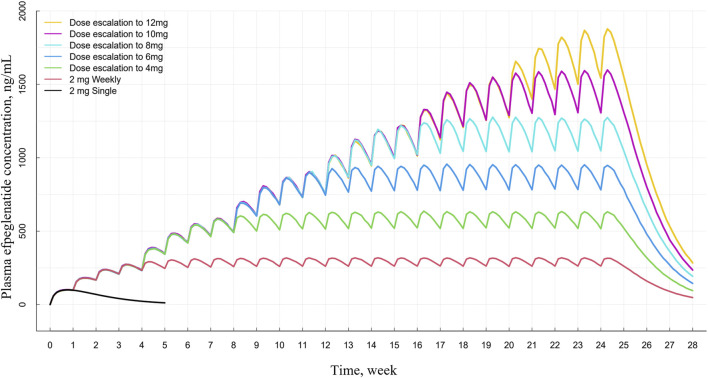
Simulated plasma efpeglenatide concentration–time profiles under stepwise dose-escalation regimens. Profiles are shown for a single 2 mg dose, repeated once-weekly 2 mg dosing, and stepwise dose escalation from 2 mg up to 4, 6, 8, 10, and 12 mg once weekly at 4-week intervals.

In the simulated concentration–time profiles of the stepwise dose-escalation regimen (2 mg–12 mg once weekly in 2 mg increments every 4 weeks), steady-state concentrations were generally achieved within 4 weeks at each dose level, and exposures increased progressively during the titration period (Figure 5).

**FIGURE 6:**
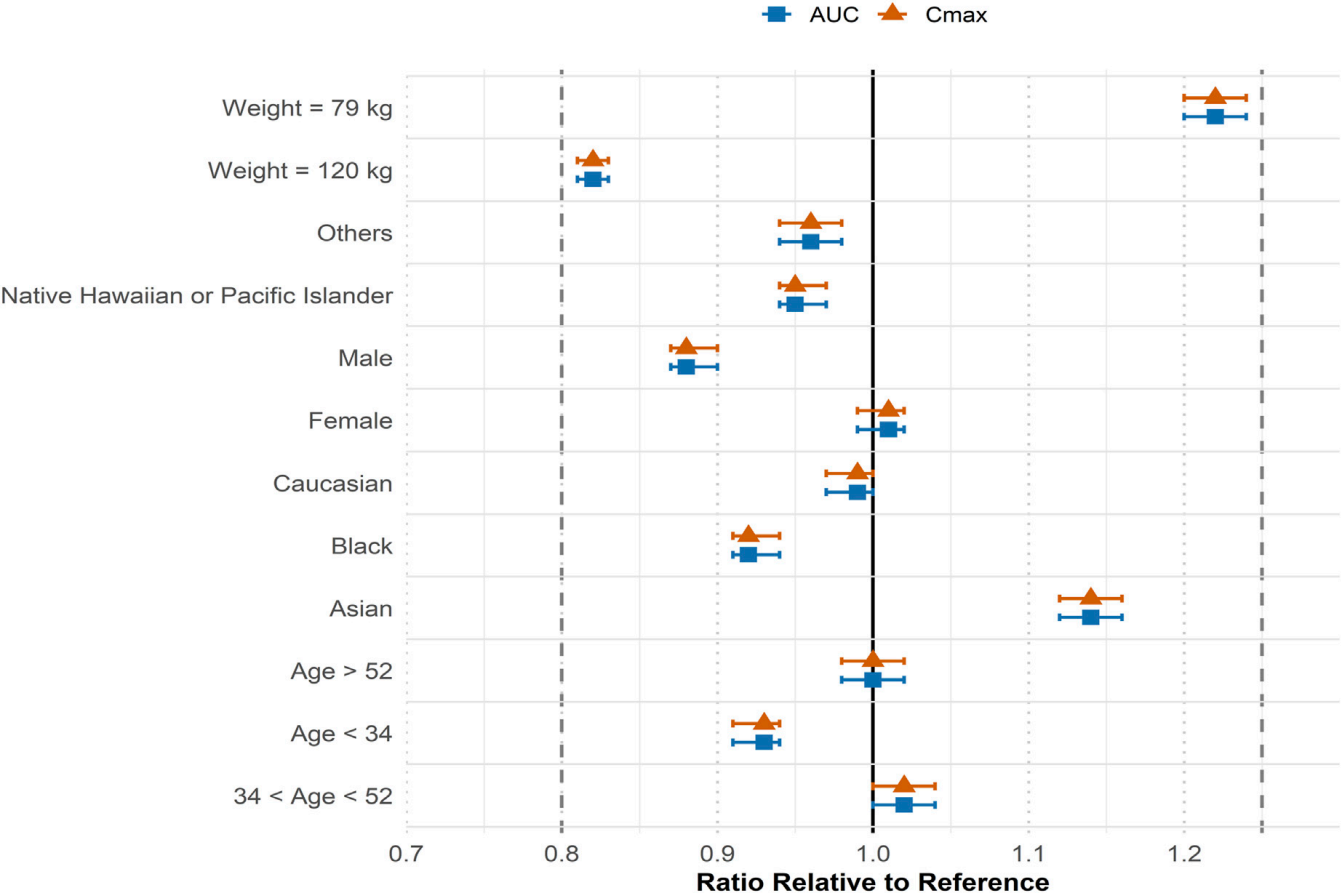
Forest plot of simulated geometric mean ratios (GMRs) and 90% confidence intervals for efpeglenatide exposure (AUC and C_max_) across covariate subgroups in obese subjects. Squares and triangles represent AUC and C_max_, respectively, relative to the reference group. Subgroup simulations were performed using representative median body weights: male (110 kg), female (95 kg), Caucasian (98 kg), Black (106 kg), Asian (83 kg), Native Hawaiian or Pacific Islander (100 kg), and Other races (101 kg). Age-based subgroup were represented by age ≤34 years (103 kg), 34–52 years (95 kg), and age ≥52 years (97 kg). For weight-based subgroups, fixed values of 79 kg (10th percentile), 96 kg (median), and 119 kg (90th percentile) were applied.

Compared with the obese median weight individual (96 kg), the 10th percentile individual (79 kg) exhibited approximately 18% lower AUC and C_max_ (GMR 0.82; 90% CI: 0.81–0.83), whereas the 90th percentile individual (120 kg) demonstrated about 20% higher exposure (GMR 1.22; 90% CI: 1.20–1.24). These results are summarized in Figure 6. Across most simulated subgroups, exposure differences remained within the commonly referenced bioequivalence range (0.80–1.25). Similarly, simulated exposures across age, sex, and race subgroups showed minimal variation from the reference, all of which were also within this range.

## 4 Discussion

Population PK analyses were conducted using pooled PK data from five phase 2 and one phase 1 clinical studies in patients with T2DM and in non-T2DM obese subjects without T2DM to characterize the PK of efpeglenatide across populations. To the best of our knowledge, this is the first population PK analysis of efpeglenatide reported in literature. The final PK model was a two-compartment linear disposition model that adequately described the observed PK profiles across studies, including the double peaks observed during the absorption phase. The second absorption peak was captured using a transit compartment approach to account for absorption delay (Savic et al., 2007).

Absorption was described by a dual-pathway structure. The fraction split between the early (direct) and delayed (transit) routes was derived from the estimated logit parameter (BIOF), corresponding to approximately 24.8% of the dose entering the depot early (BIO_A1_) and 75.2% passing through a transit chain (BIO_A2_; N = 5.52, MTT = 2.68 h) before reaching the depot; both pathways were described using the same first-order rate constant (k_a_ = 0.006 h^-1^). This model component reproduced the observed secondary rise in plasma concentrations and is interpreted as reflecting a delayed subcutaneous input captured by the transit absorption pathway. We regard this as an empirical representation of delayed absorption rather than a definitive mechanism (Richter and Jacobsen, 2014).

Apparent clearance was estimated to be low (CL/F = 0.032 L/h). This finding is aligned with the pharmacokinetic properties of Fc-fusion proteins, in which neonatal Fc receptor (FcRn)-mediated recycling limits proteolytic catabolism and prolongs systemic residence (Richter and Jacobsen, 2014). A similarly low clearance has also been reported for dulaglutide (CL = 0.059 L/h), another Fc-fusion GLP-1 receptor agonist, which is described by a two-compartment model with first order absorption and elimination (Geiser et al., 2016).

The central and peripheral volumes of distribution were estimated at 2.80 L (V_C_) and 3.96 L (V_P_), yielding a V_SS_ ≈ 6.8 L. Given that efpeglenatide is an exendin-4 analogue linked via a PEG spacer to a human IgG4 Fc, these estimates suggest distribution primarily within vascular and interstitial spaces, consistent with the ∼3–8 L V_SS_ typically observed for monoclonal antibodies (mAbs) and Fc-fusion proteins (Dirks and Meibohm, 2010; Ovacik and Lin, 2018). A similar two-compartment distribution has been reported for dulaglutide, further supporting a relatively restricted distribution pattern across Fc-fusion GLP-1 receptor agonists (Geiser et al., 2016).

In this analysis, disease status (T2DM vs. obesity) was identified as a statistically significant covariate for clearance (CL/F). The estimated typical CL/F was 0.044 L/h in T2DM and 0.032 L/h in obesity, representing a 38% higher clearance in T2DM. Consequently, systemic exposure is expected to be lower in T2DM than in obesity under the same dosing regimen. Considering the effect size and its uncertainty, the direction of this difference is consistent with observation for other GLP-1 receptor agonists; however, the effect has been described as minor for semaglutide, and interpretations for liraglutide were potentially confounded by study specific factors (Overgaard et al., 2016; Overgaard et al., 2019).

Baseline body weight was identified as a statistically significant covariate on both k_a_ and CL/F, implemented via a power function normalized to 92 kg; the exponents of −0.927 and 0.964, respectively. The negative association between body weight and k_a_ may reflect general physiological constraints on subcutaneous absorption, including reduced local blood flow, longer diffusion distances in adipose tissue, and delayed lymphatic uptake in individuals with obesity, rather than a drug-specific mechanism (European Medicines Agency, 2023). Similar effects of body weight on clearance have consistently been reported in previous population PK analyses of GLP-1 receptor agonists (Overgaard et al., 2019; Schneck and Urva, 2024). Taken together, these findings suggest that increasing body weight may be associated with slower absorption and greater clearance, which may contribute to lower systemic exposure in heavier subjects. These covariate-based differences were consistently observed in the model-based simulations. Holding other covariates constant and assuming linear pharmacokinetics, a 10% decrease in body weight increased AUC by approximately 11% and reduced apparent clearance (CL/F) by approximately 10%. A 15% decrease in body weight increased AUC by approximately 17% with a corresponding ∼15% reduction in CL/F. For a change from 96 to 79 kg (≈17.7% decrease), AUC increased by approximately 20.7% and CL/F decreased by approximately 17.1%. Because the model assumes linear elimination, changes in AUC are driven by the effect of body weight on CL/F.

Model-based simulations showed approximately dose-proportional increases in exposure across the 2–18 mg range, consistent with clinical findings for efpeglenatide and supporting the use of the model to explore PK across dosing scenarios (Yoon et al., 2020).

When stratified by disease status (T2DM vs. obesity), the simulations predicted lower exposures in T2DM than in obesity, reflecting the higher CL/F estimated for T2DM in the covariate analysis. As body weight was included as a covariate on k_a_ and CL/F, simulations translated its effect on exposure across representative body-weight percentiles in subjects with obesity. Differences in AUC and C_max_ were modest and generally fell within conventional bioequivalence bounds (0.80–1.25), indicating that routine dose adjustment based solely on baseline body weight is not warranted. Similarly, simulations using subgroup-specific median body weight for age, sex, and race showed negligible differences within the same bounds. These results indicate that while body weight influences clearance, its overall impact on systemic exposure is limited. This finding is consistent with previous population PK analyses of GLP-1 receptor agonists (Overgaard et al., 2019; Schneck and Urva, 2024).

Consistent with the weight-reducing profile of GLP-1 receptor agonists, efpeglenatide, developed for weight reduction in individuals with obesity, has been shown to induce weight loss of approximately 6%–7% in patients with T2DM (Gerstein et al., 2021). Given the body-weight effect identified in this analysis, reductions in body weight during treatment may increase systemic exposure. However model-based evaluations anchored at the median body weight among subjects with obesity (96 kg) and spanning representative lower (79 kg) and higher (120 kg) scenarios predicted that, AUC and C_max_ would remain within the commonly referenced bioequivalence range (0.80–1.25). Taken together, the PK results support no routine dose adjustment for on-treatment weight change within the prespecified weight scenarios representative of the study population.

Gastrointestinal (GI) adverse events are the most frequent class-related tolerability issues with GLP-1 receptor agonists, and stepwise dose-escalation regimens are commonly used to mitigate these events (U.S. Food and Drug Administration, 2014; Davies et al., 2022). In clinical studies, gradual titration has been shown to reduce the incidence of GI events (Fineman et al., 2004; Gough et al., 2014; Rosenstock et al., 2016). Efpeglenatide has shown an adverse event profile consistent with this drug class (Yoon et al., 2020; Gerstein et al., 2021). The model-derived effective half-life of efpeglenatide was approximately 6–7 days, consistent with clinically reported values of 5.6–7.5 days (Yoon et al., 2020). Accordingly, once-weekly dosing is expected to achieve steady state by ∼4 weeks (≈4–5 half-lives).

On this basis, a once-weekly stepwise escalation regimen (e.g., 2→12 mg in 2-mg increments every 4 weeks) was prespecified and evaluated by simulation. The design was guided by drug labels of the other GLP-1 receptor agonist and clinical guidance recommending gradual titration to improve GI tolerability. It was further supported by the model-derived effective half-life (∼6–7 days), which predicts achievement of steady state after approximately four weekly doses at each step. Together, these considerations support the clinical translatability of the model-based simulation.

Several limitations of this study should be acknowledged. First, the absorption process was represented empirically using a delayed (transit) pathway; this should be viewed as an approximation rather than a definitive mechanism and may bias apparent distribution parameters if absorption is misspecified (Dirks and Meibohm, 2010; Therapeutic Goods Administration, 2011). Second, covariate effects were assessed using baseline body weight rather than time-varying weight; incorporating longitudinal weight trajectories may better quantify the impact of on-treatment weight loss on exposure, as shown in a recent tirzepatide population-PK analysis (Schneck and Urva, 2024). In addition, because the obesity data originated from a single study, disease status is fully confounded with study; independent identification of disease versus study effects is not possible in this dataset, and the observed between-group differences should therefore be interpreted with caution. Third, the present work was limited to PK-characterization and no formal exposure–response analyses linking efpeglenatide exposure to efficacy (body weight reduction) or tolerability (GI adverse events) were conducted. Finally, exposure–response (PK–PD) analyses incorporating weight-loss effects and GI tolerability would be valuable to support selection of an optimal maintenance dose and stepwise escalation schedule. External validation with forthcoming Phase 3 clinical trial for obesity would further strengthen the generalizability and clinical applicability of these findings.

In conclusion, this analysis characterized the PK of efpeglenatide using pooled data from six clinical studies in subjects with T2DM and individuals with obesity. Disease status (T2DM vs. obesity) and body weight were identified as statistically significant covariates affecting CL/F. However, model-predicted differences associated with these covariates were minimal and not clinically meaningful, indicating that dose adjustment is not required. These results provide quantitative evidence to guide the identification of optimal dosing regimens for efpeglenatide.

## Data Availability

The original contributions presented in the study are included in the article/supplementary material, further inquiries can be directed to the corresponding author.

## Abbreviations

PK, pharmacokinetics; CL, clearance; LLOQ, lower limit of quantification; BLQ, below the limit of quantification; IIV, Interindividual variability; LRT, likelihood ratio test; GOF, goodness-of-fit; RV, residual variability; OFV, objective function value; VPC, visual predictive checks; PI, prediction interval.
